# 1-Deacet­oxy-1-oxocaesalmin

**DOI:** 10.1107/S1600536814011040

**Published:** 2014-05-17

**Authors:** Juan Feng, Jian-Long Zhang, Rong-Rong Zhang, Li-Jun Ruan, Ren-Wang Jiang

**Affiliations:** a Guangdong Province Key Laboratory of Pharmacodynamic Constituents of Traditional Chinese Medicine and New Drugs Research, Institute of Traditional Chinese Medicine and Natural Products, Jinan University, Guangzhou 510632, People’s Republic of China

## Abstract

The title compound, C_24_H_30_O_7_, is a diterpenoid isolated from the seeds of *Caesalpinia minax*. It consists of two cyclo­hexane rings (*A* and *B*), one unsaturated six-membered ring (*C*) and one furan ring (*D*). The stereochemistry of the ring junctures is *A*/*B trans* and *B*/*C trans*. Rings *A* and *B* have normal chair conformations while *C* adopts a twisted half-chair conformation due to fusion to the furan ring which is planar [r.m.s. deviation = 0.0009 (2) Å]. In the crystal, hydroxyl O—H⋯O_carbon­yl_ hydrogen bonds link the mol­ecules into a chain structure extending along the *a-*axis direction.

## Related literature   

For previous isolation of 1-deacet­oxy-1-oxocaesalmin, see: Kalauni *et al.* (2005 ▶). For the anti­viral activity of similar diterpenoids, see: Jiang *et al.* (2001 ▶). For the anti­malarial activity of similar diterpenoids, see: Kalauni *et al.* (2006 ▶). For the anti­tumor activity of similar diterpenoids, see: Ma *et al.* (2013 ▶). For the stereochemistry of caesalmin C, see: Jiang *et al.* (2001 ▶).
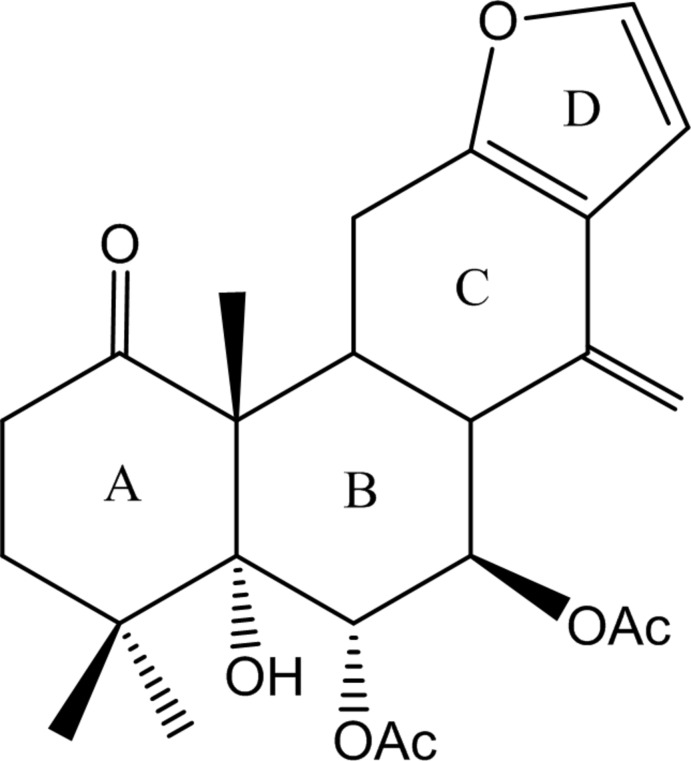


## Experimental   

### 

#### Crystal data  

C_24_H_30_O_7_
*M* *_r_* = 430.48
Orthorhombic, 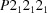

*a* = 6.7744 (1) Å
*b* = 17.2209 (4) Å
*c* = 19.1592 (5) Å
*V* = 2235.14 (8) Å^3^
*Z* = 4
Cu *K*α radiation
μ = 0.77 mm^−1^
*T* = 173 K
0.38 × 0.27 × 0.22 mm

#### Data collection  

Oxford Diffraction Gemini-S ultra Sapphire CCD diffractometer
Absorption correction: multi-scan (*CrysAlis PRO*; Agilent, 2011 ▶) *T*_min_ = 0.83, *T*_max_ = 1.00
4463 measured reflections
3080 independent reflections
2845 reflections with *I* > 2σ(*I*)
*R*_int_ = 0.020

#### Refinement  

*R*[*F* ^2^ > 2σ(*F* ^2^)] = 0.034
*wR*(*F* ^2^) = 0.087
*S* = 1.05
3080 reflections
287 parameters
H-atom parameters constrained
Δρ_max_ = 0.14 e Å^−3^
Δρ_min_ = −0.12 e Å^−3^
Absolute structure: Flack, 1983 ▶: 1031 Friedel pairs
Absolute structure parameter: −0.1 (2)

### 

Data collection: *CrysAlis PRO* (Agilent, 2011 ▶); cell refinement: *CrysAlis PRO*; data reduction: *CrysAlis PRO*; program(s) used to solve structure: *SHELXS97* (Sheldrick, 2008 ▶); program(s) used to refine structure: *SHELXL97* (Sheldrick, 2008 ▶); molecular graphics: *XP* in *SHELXTL* (Sheldrick, 2008 ▶); software used to prepare material for publication: *SHELXTL*.

## Supplementary Material

Crystal structure: contains datablock(s) I, global. DOI: 10.1107/S1600536814011040/zs2299sup1.cif

Structure factors: contains datablock(s) I. DOI: 10.1107/S1600536814011040/zs2299Isup2.hkl

CCDC reference: 1002789

Additional supporting information:  crystallographic information; 3D view; checkCIF report

## Figures and Tables

**Table 1:** Hydrogen-bond geometry (Å, °)

*D*—H⋯*A*	*D*—H	H⋯*A*	*D*⋯*A*	*D*—H⋯*A*
O2—H2⋯O1^i^	0.82	2.04	2.804 (2)	156

Symmetry code: (i) 
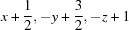
.

**Fig. 1.**
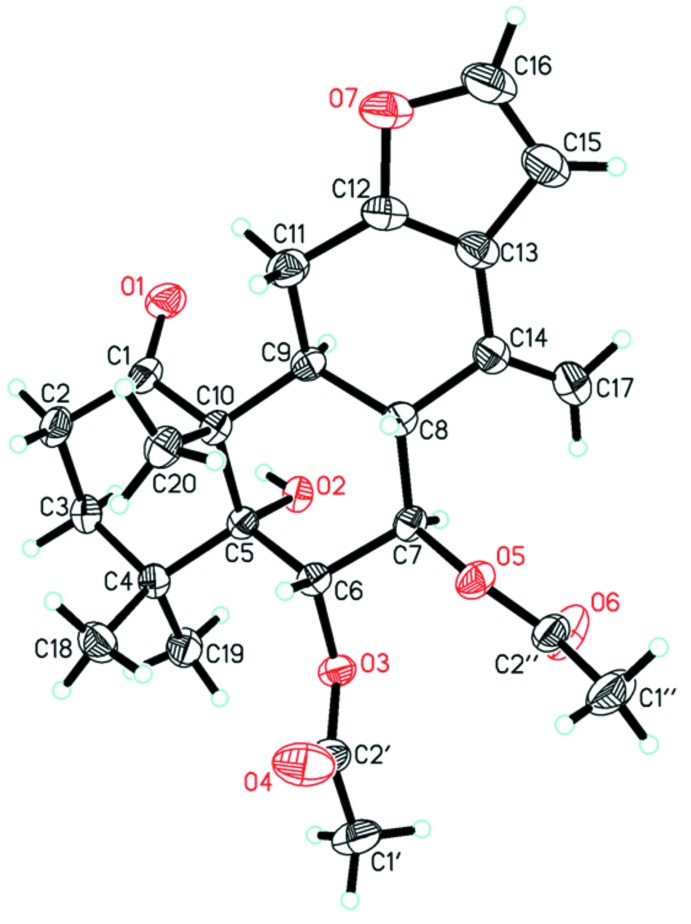
The molecular structure of the title compound showing 30% probability displacement ellipsoids.

**Fig. 2.**
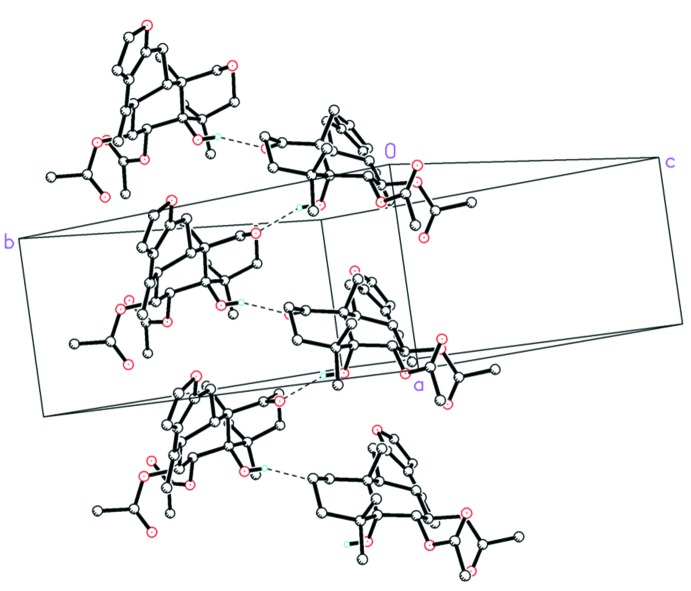
The one-dimensional chain structure in the title compound showing the intermolecular O—H···O hydrogen bonds which are represented by dashed lines. Hydrogen atoms not involved in hydrogen bonding are omitted.

## supplementary crystallographic information

### 1. Comment

1-Deacetoxy-1-oxocaesalmin is a natural diterpenoid which has been isolated from the seeds of *Caesalpinia crista* (Kalauni *et al.*, 2005). Though the biological activity of this compound was not reported, similar diterpenoids were reported to have antiviral (Jiang *et al.*, 2001), antimalarial (Kalauni *et al.*, 2006) and antitumor (Ma *et al.*, 2013) activities. *Caesalpinia minax* is a prickly scandent shrub distributing widely in the tropics and subtropics. The seeds of this plant have been used in Chinese folk medicine for the treatment of prostatitis. In order to characterize the active components, we performed a phytochemical study on the seeds of this plant. The title compound was isolated from the ethyl acetate fraction of the 95% ethanol extract followed by recrystallization from the methanol solution at room temperature. Isolation of this compound, C_24_H_30_O_7_, from *Caesalpinia minax* and *Caesalpinia crista* of the same genus indicated that it can be served as a chemotaxonomic marker for this genus.

The title compound (Fig. 1) contains two cyclohexane rings (*A* and *B*), one unsaturated six-membered ring (*C*) and one furan ring (*D*). The stereochemistry of the ring juncture is *A*/*B**trans* and *B*/*C**trans*. The cyclohexane rings *A* and *B* have normal chair conformations. The unsaturated six-membered ring (*C*) adopts a twisted half-chair conformation due to fusion to the furan ring *D* which is planar.

The absolute configuration determined for caesalmin C (Jiang *et al.*, 2001), a similar diterpenoid, was invoked, giving the assignments of the chiral centres in the molecule as shown in Fig. 1.

An intermolecular hydroxyl O2—H···O1^i^ (carbonyl) hydrogen bond (Table 1) links the molecules into a one-dimensional chain structure extending along *a* (Fig. 2).

### 2. Experimental

The dry ground seeds of *Caesalpinia minax* (5.0 kg) were refluxed with 95% EtOH. After evaporation of the solvent, the crude extract was suspended in distilled water and extracted with hexane (800 ml), ethyl acetate (800 ml) and butanol (600 ml), successively. The ethyl acetate fraction (65 g) was subjected to column chromatography over silica gel, and eluted with a cyclohexane-ethyl acetate gradient (10:1 to 0:1) to afford the title compound, which was further recrystallized from methanol at room temperature to give colorless crystals (18 mg).

### 3. Refinement

The C-bound H atoms were positioned geometrically and were included in the refinement in the riding-model approximation, with C—H = 0.96 Å (CH_3_) and *U*_iso_(H) = 1.5*U*_eq_(C); 0.97 Å (CH_2_) and *U*_iso_(H) = 1.2*U*_eq_(C); 0.98 Å (CH) and *U*_iso_(H) = 1.2*U*_eq_(C); 0.93 Å (aryl H) and *U*_iso_(H)= 1.2*U*_eq_(C); O—H = 0.82 Å and *U*_iso_(H) = 1.5*U*_eq_(O). The absolute configuration can be unambiguously assigned with reference to the known configuration of the closely related compound caesalmin C (Jiang *et al.*, 2001). [C5(*R*),C6(*S*), C7(*R*),C8(*R*),C9(*S*),C10(*R*] were assigned for the six chiral centres in the title compound using the arbitrarily named atoms employed. The Flack parameter (Flack, 1983) was refined to -0.1 (2) for 1031 Friedel pairs.

### Crystal data

**Table:**

C_24_H_30_O_7_	*F*(000) = 920
*M**_r_* = 430.48	*D*_x_ = 1.279 Mg m^−^^3^
Orthorhombic, *P*2_1_2_1_2_1_	Cu *K*α radiation, λ = 1.54184 Å
Hall symbol: P 2ac 2ab	Cell parameters from 2218 reflections
*a* = 6.7744 (1) Å	θ = 4.6–62.6°
*b* = 17.2209 (4) Å	µ = 0.77 mm^−^^1^
*c* = 19.1592 (5) Å	*T* = 173 K
*V* = 2235.14 (8) Å^3^	Block, colorless
*Z* = 4	0.38 × 0.27 × 0.22 mm

### Data collection

**Table:**

Oxford Diffraction Gemini-S ultra Sapphire CCD diffractometer	3080 independent reflections
Radiation source: fine-focus sealed tube	2845 reflections with *I* > 2σ(*I*)
Graphite monochromator	*R*_int_ = 0.020
ω scan	θ_max_ = 62.7°, θ_min_ = 4.6°
Absorption correction: multi-scan (*CrysAlis PRO*; Agilent, 2011)	*h* = −4→7
*T*_min_ = 0.83, *T*_max_ = 1.00	*k* = −19→19
4463 measured reflections	*l* = −21→13

### Refinement

**Table:**

Refinement on *F*^2^	Hydrogen site location: inferred from neighbouring sites
Least-squares matrix: full	H-atom parameters constrained
*R*[*F*^2^ > 2σ(*F*^2^)] = 0.034	*w* = 1/[σ^2^(*F*_o_^2^) + (0.0442*P*)^2^ + 0.203*P*] where *P* = (*F*_o_^2^ + 2*F*_c_^2^)/3
*wR*(*F*^2^) = 0.087	(Δ/σ)_max_ = 0.001
*S* = 1.05	Δρ_max_ = 0.14 e Å^−^^3^
3080 reflections	Δρ_min_ = −0.12 e Å^−^^3^
287 parameters	Extinction correction: *SHELXL97*, Fc^*^=kFc[1+0.001xFc^2^λ^3^/sin(2θ)]^-1/4^
0 restraints	Extinction coefficient: 0.0052 (4)
Primary atom site location: structure-invariant direct methods	Absolute structure: Flack, 1983: 1031 Friedel pairs
Secondary atom site location: difference Fourier map	Absolute structure parameter: −0.1 (2)

### Special details

**Table:**

Geometry. All e.s.d.'s (except the e.s.d. in the dihedral angle between two l.s. planes) are estimated using the full covariance matrix. The cell e.s.d.'s are taken into account individually in the estimation of e.s.d.'s in distances, angles and torsion angles; correlations between e.s.d.'s in cell parameters are only used when they are defined by crystal symmetry. An approximate (isotropic) treatment of cell e.s.d.'s is used for estimating e.s.d.'s involving l.s. planes.
Refinement. Refinement of *F*^2^ against ALL reflections. The weighted *R*-factor *wR* and goodness of fit *S* are based on *F*^2^, conventional *R*-factors *R* are based on *F*, with *F* set to zero for negative *F*^2^. The threshold expression of *F*^2^ > σ(*F*^2^) is used only for calculating *R*-factors(gt) *etc*. and is not relevant to the choice of reflections for refinement. *R*-factors based on *F*^2^ are statistically about twice as large as those based on *F*, and *R*- factors based on ALL data will be even larger.

### Fractional atomic coordinates and isotropic or equivalent isotropic displacement parameters (Å^2^)

**Table:**

	*x*	*y*	*z*	*U*_iso_*/*U*_eq_	
O1	0.6154 (2)	0.74496 (9)	0.53183 (8)	0.0547 (5)	
O2	1.00500 (18)	0.62367 (8)	0.54535 (7)	0.0354 (3)	
H2	1.0035	0.6603	0.5180	0.053*	
O3	1.0530 (2)	0.45663 (8)	0.55179 (7)	0.0420 (4)	
O4	0.8847 (3)	0.34413 (10)	0.55089 (12)	0.0795 (6)	
O5	0.9459 (2)	0.45522 (8)	0.69152 (7)	0.0443 (4)	
O6	1.2768 (3)	0.46401 (12)	0.69992 (11)	0.0765 (6)	
O7	0.3966 (3)	0.73518 (10)	0.77457 (10)	0.0655 (5)	
C1'	1.2248 (5)	0.34629 (16)	0.51680 (16)	0.0804 (9)	
H1'1	1.2095	0.2916	0.5087	0.121*	
H1'2	1.3250	0.3545	0.5515	0.121*	
H1'3	1.2627	0.3714	0.4741	0.121*	
C2'	1.0337 (4)	0.37944 (13)	0.54191 (13)	0.0546 (6)	
C1''	1.1107 (5)	0.35621 (16)	0.75089 (14)	0.0767 (9)	
H1'4	1.2374	0.3313	0.7524	0.115*	
H1'5	1.0176	0.3228	0.7279	0.115*	
H1'6	1.0666	0.3665	0.7976	0.115*	
C2''	1.1270 (4)	0.43059 (14)	0.71173 (12)	0.0519 (6)	
C1	0.6091 (3)	0.67845 (13)	0.51015 (11)	0.0431 (5)	
C2	0.5798 (4)	0.66072 (14)	0.43471 (11)	0.0518 (6)	
H2A	0.5468	0.7076	0.4092	0.062*	
H2B	0.4736	0.6236	0.4287	0.062*	
C3	0.7740 (3)	0.62687 (14)	0.40805 (11)	0.0460 (5)	
H3A	0.8746	0.6669	0.4107	0.055*	
H3B	0.7578	0.6134	0.3592	0.055*	
C4	0.8484 (3)	0.55493 (12)	0.44730 (10)	0.0395 (5)	
C5	0.8484 (3)	0.57154 (11)	0.52880 (10)	0.0330 (4)	
C6	0.8845 (3)	0.50065 (11)	0.57584 (11)	0.0352 (5)	
H6	0.7671	0.4674	0.5758	0.042*	
C7	0.9308 (3)	0.52595 (11)	0.65015 (10)	0.0355 (5)	
H7	1.0560	0.5544	0.6514	0.043*	
C8	0.7674 (3)	0.57465 (12)	0.68305 (11)	0.0367 (5)	
H8	0.6597	0.5390	0.6949	0.044*	
C9	0.6799 (3)	0.63698 (12)	0.63335 (10)	0.0369 (5)	
H9	0.7733	0.6804	0.6320	0.044*	
C10	0.6501 (3)	0.60885 (11)	0.55740 (11)	0.0356 (5)	
C11	0.4861 (3)	0.66772 (15)	0.66522 (12)	0.0518 (6)	
H11A	0.4424	0.7135	0.6401	0.062*	
H11B	0.3839	0.6284	0.6618	0.062*	
C12	0.5223 (4)	0.68734 (13)	0.73927 (12)	0.0486 (6)	
C13	0.6726 (4)	0.66413 (12)	0.77927 (12)	0.0495 (6)	
C14	0.8301 (3)	0.61460 (12)	0.75107 (11)	0.0426 (5)	
C15	0.6410 (5)	0.70010 (16)	0.84602 (14)	0.0683 (8)	
H15	0.7208	0.6954	0.8853	0.082*	
C16	0.4758 (5)	0.74136 (17)	0.84077 (15)	0.0755 (9)	
H16	0.4211	0.7705	0.8768	0.091*	
C17	1.0046 (4)	0.60919 (15)	0.78090 (12)	0.0564 (6)	
H17A	1.0315	0.6373	0.8212	0.068*	
H17B	1.1010	0.5773	0.7616	0.068*	
C18	0.7257 (4)	0.48423 (14)	0.42442 (13)	0.0557 (6)	
H18A	0.5886	0.4983	0.4227	0.084*	
H18B	0.7438	0.4427	0.4573	0.084*	
H18C	0.7680	0.4676	0.3790	0.084*	
C19	1.0596 (3)	0.54143 (16)	0.41966 (12)	0.0532 (6)	
H19A	1.0580	0.5410	0.3696	0.080*	
H19B	1.1079	0.4925	0.4365	0.080*	
H19C	1.1443	0.5824	0.4358	0.080*	
C20	0.4714 (3)	0.55316 (14)	0.55402 (12)	0.0469 (6)	
H20A	0.3513	0.5828	0.5540	0.070*	
H20B	0.4731	0.5194	0.5939	0.070*	
H20C	0.4786	0.5227	0.5121	0.070*	

### Atomic displacement parameters (Å^2^)

**Table:**

	*U*^11^	*U*^22^	*U*^33^	*U*^12^	*U*^13^	*U*^23^
O1	0.0695 (11)	0.0417 (9)	0.0530 (9)	0.0148 (8)	0.0001 (9)	0.0065 (8)
O2	0.0329 (7)	0.0355 (7)	0.0378 (7)	−0.0067 (6)	−0.0040 (6)	0.0067 (6)
O3	0.0434 (8)	0.0341 (7)	0.0486 (8)	0.0060 (6)	0.0001 (7)	−0.0012 (7)
O4	0.0966 (15)	0.0402 (10)	0.1016 (15)	−0.0114 (11)	0.0139 (13)	−0.0059 (10)
O5	0.0491 (8)	0.0377 (8)	0.0461 (8)	0.0054 (7)	0.0038 (7)	0.0120 (7)
O6	0.0493 (10)	0.0942 (15)	0.0858 (14)	0.0124 (10)	−0.0027 (10)	0.0337 (13)
O7	0.0731 (12)	0.0582 (10)	0.0650 (11)	0.0128 (9)	0.0214 (10)	−0.0058 (9)
C1'	0.097 (2)	0.0611 (18)	0.083 (2)	0.0382 (17)	−0.0011 (18)	−0.0062 (16)
C2'	0.0810 (18)	0.0343 (12)	0.0484 (13)	0.0089 (13)	−0.0038 (13)	0.0009 (11)
C1''	0.097 (2)	0.0647 (18)	0.0682 (17)	0.0341 (17)	0.0125 (17)	0.0269 (14)
C2''	0.0611 (15)	0.0550 (14)	0.0397 (12)	0.0224 (13)	0.0053 (12)	0.0086 (11)
C1	0.0332 (10)	0.0490 (13)	0.0469 (12)	0.0080 (10)	−0.0011 (10)	0.0077 (11)
C2	0.0526 (13)	0.0573 (14)	0.0456 (13)	0.0102 (12)	−0.0103 (11)	0.0094 (11)
C3	0.0488 (13)	0.0519 (13)	0.0373 (11)	0.0008 (11)	−0.0069 (10)	0.0029 (11)
C4	0.0391 (11)	0.0425 (12)	0.0368 (11)	0.0004 (10)	−0.0038 (9)	−0.0005 (10)
C5	0.0293 (10)	0.0306 (10)	0.0390 (11)	−0.0033 (8)	−0.0031 (8)	0.0011 (9)
C6	0.0320 (10)	0.0317 (10)	0.0418 (11)	−0.0012 (9)	0.0007 (9)	0.0019 (9)
C7	0.0383 (11)	0.0312 (10)	0.0372 (11)	−0.0021 (9)	0.0001 (9)	0.0091 (9)
C8	0.0369 (10)	0.0322 (10)	0.0409 (11)	−0.0016 (9)	0.0028 (9)	0.0053 (9)
C9	0.0370 (11)	0.0338 (11)	0.0400 (11)	0.0015 (9)	0.0010 (9)	0.0028 (9)
C10	0.0305 (10)	0.0366 (11)	0.0396 (11)	0.0010 (9)	−0.0018 (9)	0.0046 (9)
C11	0.0468 (13)	0.0568 (14)	0.0517 (13)	0.0116 (11)	0.0076 (11)	0.0038 (12)
C12	0.0574 (13)	0.0378 (12)	0.0508 (13)	0.0029 (11)	0.0158 (12)	−0.0007 (10)
C13	0.0698 (16)	0.0348 (11)	0.0438 (12)	−0.0012 (12)	0.0095 (12)	0.0010 (10)
C14	0.0543 (13)	0.0367 (11)	0.0368 (11)	−0.0029 (10)	0.0026 (10)	0.0056 (9)
C15	0.104 (2)	0.0528 (15)	0.0483 (14)	0.0038 (17)	0.0083 (16)	−0.0071 (12)
C16	0.108 (2)	0.0606 (17)	0.0575 (17)	0.0113 (18)	0.0218 (18)	−0.0094 (14)
C17	0.0662 (16)	0.0608 (15)	0.0423 (12)	−0.0050 (13)	−0.0025 (12)	−0.0024 (12)
C18	0.0631 (15)	0.0510 (14)	0.0529 (14)	−0.0068 (12)	−0.0073 (13)	−0.0088 (12)
C19	0.0477 (13)	0.0703 (16)	0.0417 (12)	0.0091 (13)	0.0033 (11)	−0.0005 (12)
C20	0.0297 (10)	0.0550 (14)	0.0559 (14)	−0.0018 (10)	−0.0022 (10)	0.0013 (12)

### Geometric parameters (Å, º)

**Table:**

O1—C1	1.219 (3)	C6—C7	1.522 (3)
O2—C5	1.426 (2)	C6—H6	0.9800
O2—H2	0.8200	C7—C8	1.525 (3)
O3—C2'	1.349 (3)	C7—H7	0.9800
O3—C6	1.445 (2)	C8—C14	1.534 (3)
O4—C2'	1.191 (3)	C8—C9	1.552 (3)
O5—C2''	1.355 (3)	C8—H8	0.9800
O5—C7	1.457 (2)	C9—C11	1.542 (3)
O6—C2''	1.188 (3)	C9—C10	1.547 (3)
O7—C12	1.364 (3)	C9—H9	0.9800
O7—C16	1.381 (4)	C10—C20	1.546 (3)
C1'—C2'	1.494 (4)	C11—C12	1.479 (3)
C1'—H1'1	0.9600	C11—H11A	0.9700
C1'—H1'2	0.9600	C11—H11B	0.9700
C1'—H1'3	0.9600	C12—C13	1.336 (3)
C1''—C2''	1.489 (3)	C13—C15	1.437 (3)
C1''—H1'4	0.9600	C13—C14	1.469 (3)
C1''—H1'5	0.9600	C14—C17	1.316 (3)
C1''—H1'6	0.9600	C15—C16	1.329 (4)
C1—C2	1.491 (3)	C15—H15	0.9300
C1—C10	1.527 (3)	C16—H16	0.9300
C2—C3	1.527 (3)	C17—H17A	0.9300
C2—H2A	0.9700	C17—H17B	0.9300
C2—H2B	0.9700	C18—H18A	0.9600
C3—C4	1.534 (3)	C18—H18B	0.9600
C3—H3A	0.9700	C18—H18C	0.9600
C3—H3B	0.9700	C19—H19A	0.9600
C4—C18	1.538 (3)	C19—H19B	0.9600
C4—C19	1.543 (3)	C19—H19C	0.9600
C4—C5	1.587 (3)	C20—H20A	0.9600
C5—C6	1.537 (3)	C20—H20B	0.9600
C5—C10	1.587 (3)	C20—H20C	0.9600
			
C5—O2—H2	109.5	C7—C8—C14	113.38 (17)
C2'—O3—C6	119.02 (18)	C7—C8—C9	113.82 (16)
C2''—O5—C7	118.75 (17)	C14—C8—C9	108.46 (16)
C12—O7—C16	105.0 (2)	C7—C8—H8	106.9
C2'—C1'—H1'1	109.5	C14—C8—H8	106.9
C2'—C1'—H1'2	109.5	C9—C8—H8	106.9
H1'1—C1'—H1'2	109.5	C11—C9—C10	111.64 (17)
C2'—C1'—H1'3	109.5	C11—C9—C8	108.64 (17)
H1'1—C1'—H1'3	109.5	C10—C9—C8	114.23 (16)
H1'2—C1'—H1'3	109.5	C11—C9—H9	107.3
O4—C2'—O3	124.4 (2)	C10—C9—H9	107.3
O4—C2'—C1'	125.8 (2)	C8—C9—H9	107.3
O3—C2'—C1'	109.7 (2)	C1—C10—C20	108.65 (16)
C2''—C1''—H1'4	109.5	C1—C10—C9	109.61 (16)
C2''—C1''—H1'5	109.5	C20—C10—C9	109.63 (16)
H1'4—C1''—H1'5	109.5	C1—C10—C5	105.48 (16)
C2''—C1''—H1'6	109.5	C20—C10—C5	113.41 (16)
H1'4—C1''—H1'6	109.5	C9—C10—C5	109.94 (15)
H1'5—C1''—H1'6	109.5	C12—C11—C9	108.5 (2)
O6—C2''—O5	124.6 (2)	C12—C11—H11A	110.0
O6—C2''—C1''	125.2 (2)	C9—C11—H11A	110.0
O5—C2''—C1''	110.3 (2)	C12—C11—H11B	110.0
O1—C1—C2	121.8 (2)	C9—C11—H11B	110.0
O1—C1—C10	121.94 (19)	H11A—C11—H11B	108.4
C2—C1—C10	115.99 (19)	C13—C12—O7	111.8 (2)
C1—C2—C3	106.73 (18)	C13—C12—C11	127.5 (2)
C1—C2—H2A	110.4	O7—C12—C11	120.7 (2)
C3—C2—H2A	110.4	C12—C13—C15	105.6 (2)
C1—C2—H2B	110.4	C12—C13—C14	121.1 (2)
C3—C2—H2B	110.4	C15—C13—C14	133.3 (2)
H2A—C2—H2B	108.6	C17—C14—C13	122.3 (2)
C2—C3—C4	115.30 (18)	C17—C14—C8	125.9 (2)
C2—C3—H3A	108.4	C13—C14—C8	111.82 (19)
C4—C3—H3A	108.4	C16—C15—C13	106.8 (3)
C2—C3—H3B	108.4	C16—C15—H15	126.6
C4—C3—H3B	108.4	C13—C15—H15	126.6
H3A—C3—H3B	107.5	C15—C16—O7	110.8 (2)
C3—C4—C18	108.77 (17)	C15—C16—H16	124.6
C3—C4—C19	104.95 (18)	O7—C16—H16	124.6
C18—C4—C19	106.51 (19)	C14—C17—H17A	120.0
C3—C4—C5	109.67 (16)	C14—C17—H17B	120.0
C18—C4—C5	115.01 (18)	H17A—C17—H17B	120.0
C19—C4—C5	111.41 (16)	C4—C18—H18A	109.5
O2—C5—C6	104.55 (15)	C4—C18—H18B	109.5
O2—C5—C10	107.36 (14)	H18A—C18—H18B	109.5
C6—C5—C10	104.72 (15)	C4—C18—H18C	109.5
O2—C5—C4	109.38 (15)	H18A—C18—H18C	109.5
C6—C5—C4	115.69 (16)	H18B—C18—H18C	109.5
C10—C5—C4	114.39 (16)	C4—C19—H19A	109.5
O3—C6—C7	106.61 (16)	C4—C19—H19B	109.5
O3—C6—C5	110.82 (15)	H19A—C19—H19B	109.5
C7—C6—C5	110.73 (16)	C4—C19—H19C	109.5
O3—C6—H6	109.5	H19A—C19—H19C	109.5
C7—C6—H6	109.5	H19B—C19—H19C	109.5
C5—C6—H6	109.5	C10—C20—H20A	109.5
O5—C7—C6	106.51 (15)	C10—C20—H20B	109.5
O5—C7—C8	106.62 (15)	H20A—C20—H20B	109.5
C6—C7—C8	113.25 (17)	C10—C20—H20C	109.5
O5—C7—H7	110.1	H20A—C20—H20C	109.5
C6—C7—H7	110.1	H20B—C20—H20C	109.5
C8—C7—H7	110.1		
			
C6—O3—C2'—O4	0.9 (4)	C2—C1—C10—C20	−61.1 (2)
C6—O3—C2'—C1'	179.70 (19)	O1—C1—C10—C9	4.7 (3)
C7—O5—C2''—O6	2.4 (3)	C2—C1—C10—C9	179.16 (18)
C7—O5—C2''—C1''	−177.83 (18)	O1—C1—C10—C5	−113.6 (2)
O1—C1—C2—C3	111.0 (2)	C2—C1—C10—C5	60.8 (2)
C10—C1—C2—C3	−63.5 (2)	C11—C9—C10—C1	69.0 (2)
C1—C2—C3—C4	56.6 (2)	C8—C9—C10—C1	−167.21 (17)
C2—C3—C4—C18	76.1 (2)	C11—C9—C10—C20	−50.1 (2)
C2—C3—C4—C19	−170.24 (18)	C8—C9—C10—C20	73.6 (2)
C2—C3—C4—C5	−50.5 (2)	C11—C9—C10—C5	−175.44 (17)
C3—C4—C5—O2	−72.5 (2)	C8—C9—C10—C5	−51.7 (2)
C18—C4—C5—O2	164.53 (17)	O2—C5—C10—C1	70.52 (18)
C19—C4—C5—O2	43.2 (2)	C6—C5—C10—C1	−178.73 (16)
C3—C4—C5—C6	169.76 (16)	C4—C5—C10—C1	−51.0 (2)
C18—C4—C5—C6	46.8 (2)	O2—C5—C10—C20	−170.70 (15)
C19—C4—C5—C6	−74.5 (2)	C6—C5—C10—C20	−60.0 (2)
C3—C4—C5—C10	47.9 (2)	C4—C5—C10—C20	67.7 (2)
C18—C4—C5—C10	−75.0 (2)	O2—C5—C10—C9	−47.6 (2)
C19—C4—C5—C10	163.66 (18)	C6—C5—C10—C9	63.17 (19)
C2'—O3—C6—C7	110.59 (19)	C4—C5—C10—C9	−169.14 (16)
C2'—O3—C6—C5	−128.84 (19)	C10—C9—C11—C12	175.38 (18)
O2—C5—C6—O3	−72.25 (18)	C8—C9—C11—C12	48.5 (2)
C10—C5—C6—O3	174.99 (14)	C16—O7—C12—C13	−0.1 (3)
C4—C5—C6—O3	48.1 (2)	C16—O7—C12—C11	−179.3 (2)
O2—C5—C6—C7	45.8 (2)	C9—C11—C12—C13	−17.5 (3)
C10—C5—C6—C7	−66.91 (19)	C9—C11—C12—O7	161.5 (2)
C4—C5—C6—C7	166.20 (16)	O7—C12—C13—C15	0.0 (3)
C2''—O5—C7—C6	105.7 (2)	C11—C12—C13—C15	179.0 (2)
C2''—O5—C7—C8	−133.07 (19)	O7—C12—C13—C14	−177.6 (2)
O3—C6—C7—O5	−64.06 (18)	C11—C12—C13—C14	1.4 (4)
C5—C6—C7—O5	175.31 (15)	C12—C13—C14—C17	159.7 (2)
O3—C6—C7—C8	179.06 (15)	C15—C13—C14—C17	−17.1 (4)
C5—C6—C7—C8	58.4 (2)	C12—C13—C14—C8	−17.9 (3)
O5—C7—C8—C14	75.5 (2)	C15—C13—C14—C8	165.3 (2)
C6—C7—C8—C14	−167.69 (16)	C7—C8—C14—C17	−0.5 (3)
O5—C7—C8—C9	−159.89 (16)	C9—C8—C14—C17	−127.9 (2)
C6—C7—C8—C9	−43.1 (2)	C7—C8—C14—C13	177.05 (17)
C7—C8—C9—C11	166.11 (18)	C9—C8—C14—C13	49.6 (2)
C14—C8—C9—C11	−66.7 (2)	C12—C13—C15—C16	0.2 (3)
C7—C8—C9—C10	40.8 (2)	C14—C13—C15—C16	177.4 (3)
C14—C8—C9—C10	167.97 (16)	C13—C15—C16—O7	−0.3 (3)
O1—C1—C10—C20	124.5 (2)	C12—O7—C16—C15	0.2 (3)

### Hydrogen-bond geometry (Å, º)

**Table:**

*D*—H···*A*	*D*—H	H···*A*	*D*···*A*	*D*—H···*A*
O2—H2···O1^i^	0.82	2.04	2.804 (2)	156

Symmetry code: (i) *x*+1/2, −*y*+3/2, −*z*+1.
